# Accuracy of diagnostic registers and management of chronic obstructive pulmonary disease: the Devon primary care audit

**DOI:** 10.1186/1465-9921-9-62

**Published:** 2008-08-18

**Authors:** Rupert CM Jones, Maria Dickson-Spillmann, Martin JC Mather, Dawn Marks, Bryanie S Shackell

**Affiliations:** 1 Respiratory Research Unit, Peninsula Medical School, 1 Davy Road, Plymouth, UK; 2 The Waterside Practice, St. Brannocks Road, Ilfracombe, UK

## Abstract

**Background:**

Guidelines on COPD diagnosis and management encourage primary care physicians to detect the disease at an early stage and to treat patients according to their condition and needs. Problems in guideline implementation include difficulties in diagnosis, using spirometry and the disputed role of reversibility testing. These lead to inaccurate diagnostic registers and inadequacy of administered treatments. This study represents an audit of COPD diagnosis and management in primary care practices in Devon.

**Methods:**

Six hundred and thirty two patients on COPD registers in primary care practices were seen by a visiting Respiratory Specialist Nurse. Diagnoses were made according to the NICE guidelines. Reversibility testing was carried out either routinely or based on clinical indication in two sub-samples. Dyspnoea was assessed. Data were entered into a novel IT-based software which computed guideline-based treatment recommendations. Current and recommended treatments were compared.

**Results:**

Five hundred and eighty patients had spirometry. Diagnoses of COPD were confirmed in 422 patients (73%). Thirty nine patients were identified as asthma only, 94 had normal spirometry, 23 were restrictive and 2 had a cardiac disorder. Reversibility testing changed diagnosis of 11% of patients with airflow obstruction, and severity grading in 18%. Three quarters of patients with COPD had been offered practical help with smoking cessation. Short and long-acting anticholinergics and long acting beta-2 agonists had been under-prescribed; in 15–18% of patients they were indicated but not received. Inhaled steroids had been over-prescribed (recommended in 17%; taken by 60%), whereas only 4% of patients with a chronic productive cough were receiving mucolytics. Pulmonary rehabilitation was not available in some areas and was under-used in other areas.

**Conclusion:**

Diagnostic registers of COPD in primary care contain mistakes leading to inaccurate prevalence estimates and inappropriate treatment decisions. Use of pre-bronchodilator readings for diagnosis overestimates the prevalence and severity in a significant minority, thus post bronchodilator readings should be used. Management of stable COPD does often not correspond to guidelines. The IT system used in this study has the potential to improve diagnosis and management of COPD in primary care.

## Background

Chronic obstructive pulmonary disease (COPD) is a growing burden to patients and the National Health Service. Disease progression is preventable by early detection combined with smoking cessation [1,2]. The annual costs are estimated at £1,639 per patient, 54% of this is for unscheduled care relating to exacerbations [3]. In primary care, earlier diagnosis and the use of interventions aimed at preventing exacerbations and delaying the progression of the disease may be the best way to tackle these costs [4].

The guidelines of the National Institute for Health and Clinical Excellence (NICE) on COPD diagnosis and management [5] are clear and comprehensive. However, major problems regarding the practical implementation of these guidelines have been identified that may affect the accuracy of COPD diagnosis and treatment.

Spirometry is a fundamental component of COPD diagnosis [5,6], and can be performed accurately in primary care [7]. However, some studies have found major problems with existing spirometry [8-11]. Up to one third of practices had no spirometer, in addition to which practice nurses lacked training and support in performing spirometry and interpreting the results [9].

At present, neither the NICE nor the GOLD guidelines recommend reversibility testing in the diagnosis of COPD. Post-bronchodilator spirometry is necessary for diagnosis of COPD according to the GOLD guidelines [6], but not the NICE guidelines [5].

Reversibility testing using bronchodilators affects the frequency of diagnoses of COPD [12,13]. Prevalence of COPD was 27% lower using post-bronchodilator spirometry compared to spirometry without bronchodilator [12].

Reversibility testing may also influence treatment decisions. For example, based on a study that used post-bronchodilator readings [14], inhaled steroids are recommended for patients who have a forced expiratory volume in one second (FEV1) ≤ 50% predicted and two or more exacerbations per year [5]. Thus, failure to use bronchodilators may cause some patients to be given treatment they do not need.

There is widespread evidence of poor adherence to guidelines in the management of COPD [15-20]. Studies in primary care have demonstrated problems with provision of most treatments including medications, vaccination, smoking cessation advice and referral to pulmonary rehabilitation. For example, 25% of general practitioners (GPs) systematically prescribed inhaled corticosteroids to patients with severe COPD, but nearly half of them were unaware of the guideline-based indication for steroids [19]. In the United Kingdom, pulmonary rehabilitation is available to only 2% of those who need it [21]. Provision of advice on smoking cessation is also poor. Hyland et al. [22] reported that more than one third of current smokers with COPD had not been offered help with smoking cessation such as referral to a smoking cessation clinic or pharmacological therapy.

At present, the quality of diagnosis, assessment and management of COPD is variable and does not always relate to the available knowledge about the disease and the burden it represents. The aims of this study were to assess the diagnostic accuracy of COPD registers in general practice and to evaluate guideline adherence in the treatment of stable COPD.

## Methods

### Recruitment procedure

All practices in the Plymouth area were invited to take part. The first 13 practices to respond were included. Three North Devon practices agreed to participate.

Project nurses (Respiratory Specialist Nurses trained and experienced in primary care management of COPD including spirometry) collected data from February 2005 to March 2006. Practice registers were electronically searched using diagnostic codes for COPD. Exclusion criteria were: serious co-morbidity affecting the patient's ability to take part or to perform spirometry, or inability to attend the surgery. Records of all patients on the COPD register were examined and those with coding errors or normal spirometry were excluded. The remaining suitable patients were invited to an appointment with the project nurse.

### Audit procedure

The South West Multicentre Research Ethics Committee confirmed that as a service evaluation, formal research ethics approval was not required for the audit. Patients were informed about the study and confidentiality issues. Patient consent was obtained to collect and analyse the data using an electronic consent form approved by the NHS information security and registration authority.

Patient assessment was based on a custom written software package. Demographic and clinical data were entered by the project nurse (Table 1) and questionnaire data were entered by the patients. Spirometry was performed without bronchodilator therapy in all patients according to European Respiratory Society and American Thoracic Society standards [23]. The spirometers used were a MicroLab ML3500 (Plymouth) and a MicroLab ML3300 (North Devon). Reversibility testing was performed using 400–800 mcg salbutamol via a large volume spacer, with spirometry repeated after 15 minutes. In Plymouth, reversibility testing was carried out only if clinically indicated to separate asthma from COPD; in North Devon, reversibility testing was performed on all patients.

**Table 1:** Data gathered by the COPD assessment software (questionnaires not shown)

Demographic data
Weight, height and body mass index (BMI)
Spirometry results: FEV1 and FVC (litres and % of predicted) pre and post bronchodilator
Number of exacerbations in previous year
Number of antibiotics for respiratory tract infections in the previous 12 months
Number of oral steroid courses in the previous 12 months
Number of out of hours visits in the previous 12 months
Number of attendances of Accident and Emergency (A & E) in the previous 12 months
Number of hospital admissions in the previous 12 months
Number of bed days in the previous 12 months
Whether patient had an x-ray at the time of diagnosis or in the previous 5 years
Vaccination status (pneumococcus and influenza)
Current COPD medication: short and long acting bronchodilators, inhaled corticosteroids, mucolytics, other prescribed medications
Inhaler technique: good, moderate, poor
Use of nebuliser
Smoking history: age smoking started, date of cessation, average number of cigarettes per day
Whether patient had undergone smoking cessation treatment
Oxygen assessment and therapy, cor pulmonale, cyanosis
Pulse oximetry value
Attendance of specialist services: respiratory specialist nurse/physiotherapy in the previous 2 years, chest clinic in the previous 5 years, pulmonary rehabilitation ever

After clinical assessments, patients completed on-screen questionnaires. One question was seen at a time, and each possible response was numbered. Patients selected their response and pressed the appropriate number on the keyboard.

Questionnaires included the Medical Research Council (MRC) Dyspnoea Scale [24], the Clinical COPD Questionnaire (CCQ) [25] and the Lung Information Needs Questionnaire (LINQ) [22].

Patients were also asked about their sputum production, (in order to assess the need for mucolytic therapy), and whether their symptoms were relieved by short-acting bronchodilators, (to help assess whether they were receiving adequate bronchodilator treatment).

Following completion of clinical assessments and questionnaires, the program automatically determined the patient's recommended treatment according to the NICE guidelines. For example, inhaled steroids were recommended if FEV1 was less than 50% predicted and if the patient had had two or more exacerbations during the previous year. Recommendations for bronchodilator treatment were based on the nurse's clinical judgement and whether their current therapy relieved their symptoms rather than computer logic. Mucolytic therapy was recommended to be considered if the patient had a chronic productive cough.

At the end of the assessment, the nurse reviewed the collected data and confirmed the diagnosis. The computer then produced two separate reports summarising the results of the assessments; a clinical report for the GP or practice nurse and a report in layman's terms for the patient.

### The COPD assessment software

The software was revised and improved during the process of the audit in accordance with feedback from patients, project nurses and primary care clinicians. Revisions included requesting data on treatment according to guidelines, shortening GP reports, and simplifying patient reports. As a result of this, there are varying numbers of patients in the statistical analyses.

### Diagnostic criteria

Spirometric results were interpreted according to the NICE recommendations [5]. If reversibility testing was applied, diagnosis was based on post-bronchodilator values. A diagnosis of restriction was given if the FEV1 (% predicted) was less than 80% and the ratio of FEV1 to forced vital capacity (FVC) was greater or equal to 0.7.

Asthma was diagnosed on the basis of both spirometric and clinical features such as the patient's history and family history which were obtained from the patient and examination of their primary care records. Asthma was confirmed if a patient with airflow obstruction returned to normal spirometric values or if a large change in FEV1 (> 400 ml) was observed in response to bronchodilators. Some patients had clinical features of both asthma and COPD as described in the NICE guidelines.

### Data analysis

Data was transferred into SPSS (Version 14.0, SPSS Inc., Chicago IL). Descriptive statistics and cross-tables were used to evaluate the accuracy of diagnostic registers and guideline adherence in treatment. If applicable, t tests or chi square tests were used to analyse between-group differences (α = 0.05).

## Results

### Characteristics of invited and excluded patients

Of the 841 patients invited for assessment, 619 were seen. Thirty-nine patients (6%) were unable to perform spirometry.

To examine the possibility of selection bias, a sub-sample of 288 patients on the COPD register were examined in more detail. Of these patients, 47 (16%) were excluded as they were unable to attend the practice; the remaining 241 patients were invited. Of these, 43 (18%) patients declined. No differences in age and gender were seen between those who attended and those who declined (Table 2).

**Table 2:** Age and gender of North Devon patients who declined and who were seen

	Declined	Attended	Significance
Age	66.4 (11.3)	68.1 (10.0)	t(241) = -0.997, p = 0.320
Gender (Males)	18/43 (42%)	127/198 (64%)	X^2^(1) = 0.27, p = 0.603

### Accuracy of diagnostic registers

Of the 580 patients who underwent spirometry, 88 (15%) patients had normal values, 456 (79%) showed obstruction, and 36 (6%) showed restriction.

**Figure 1:**
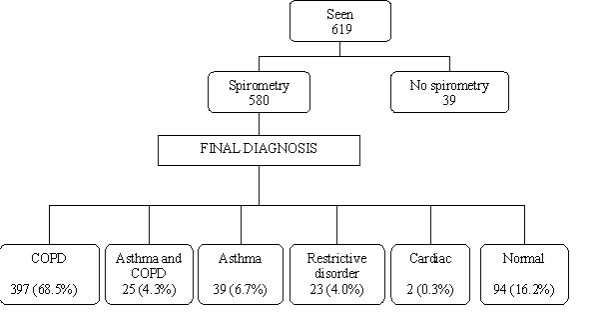
Final diagnoses in the Plymouth COPD audit sample.

Final diagnoses based on spirometric and clinical features are shown in Figure 1. Of 580 patients who had spirometry, 158 (27%) were found not to have COPD, as per the NICE guidelines. Of all 422 patients who received the final diagnosis of COPD, 25 patients (6%) had both asthma and COPD.

### Reversibility testing in patients with airflow obstruction

Airway obstruction was found in 456 patients and reversibility testing was undertaken in 232 (51%). In 140 patients reversibility testing was performed routinely (North Devon patients) and in 92 patients as clinically indicated to exclude asthma from COPD (Plymouth patients).

Reversibility testing led to a change in diagnosis for 25/232 (11%) patients. Where reversibility testing was performed routinely, the diagnosis changed in 7/140 (5%), with five being changed from a diagnosis of obstruction to normal, and two changing from a diagnosis of obstruction to one of restriction. Where reversibility testing was performed as clinically indicated, diagnostic changes occurred in 18/92 (20%) patients, with 14 changing from a diagnosis of obstruction to normal, and four changing from a diagnosis of obstruction to restriction.

As regards the magnitude of change in FEV1, 162 patients (70%) changed by up to 200 ml, 58 patients (25%) changed by 200–400 ml, and in 12 patients (5%) the change was larger than 400 ml.

In patients who showed a small change of FEV1 (up to 200 ml), diagnosis was changed in 8/162 (5%). In patients with a change up to 400 ml, diagnosis was changed in 12/58 (21%), and in those with a change of more than 400 ml, diagnosis was changed in 5/12 (42%). In the large majority of cases the diagnosis was changed from COPD to asthma. Five of the 12 patients with more than 400 ml changes in FEV1 through reversibility testing met the criteria for asthma based on post bronchodilator readings alone.

The severity grading of airflow obstruction based on pre-bronchodilator readings changed after bronchodilator in 41/232 (18%) patients. Thirty-one patients changed from moderate to mild disease, ten from severe to moderate. Three patients had lower spirometry readings after bronchodilators and in these cases their pre-bronchodilator readings were used to classify severity grading.

### Characteristics of patients with confirmed COPD

The cohort of 422 patients with confirmed COPD was examined further. The mean FEV1 was 1.25 (SD 0.44) litres and mean FEV1% predicted was 50.3% (SD 14.3). Based on the NICE guidelines, 227 (54%) patients had mild COPD, 159 (38%) patients had moderate COPD, and 36 (9%) patients had severe COPD.

The mean (SD) age was 69.2 (8.7) years. Body mass index was low (< 18.5) in 26/420 (6%); 32% were normal and 62% were overweight (BMI > 25).

One hundred and thirty-seven patients (33%) were current smokers with a mean consumption of 20 cigarettes per day and an exposure of 48 pack years; 276 (65%) were ex-smokers with a mean exposure of 43 pack years and ten patients (2%) had never smoked. Of the current smokers, 76% had been offered help to quit smoking, either with nicotine gum or patches, or by referral to a smoking cessation clinic.

Exacerbations occurred in all grades of airflow obstruction. Patients with severe airflow obstruction had received more steroid courses in the previous year than patients with milder obstruction. Interestingly, healthcare consumption (out of hours visits, accident and emergency attendances, bed days spent in hospital) was not particularly skewed towards patients with severe airflow obstruction (Table 3). Respiratory failure or cor pulmonale was noted in five patients.

**Table 3:** Frequency of exacerbations, antibiotic and steroid courses and healthcare consumption in patients with confirmed COPD

Number in previous year of:	Mild (N = 226)	Moderate (N = 158)	Severe (N = 36)	All (N = 420)
Exacerbations	1.3 (1.7)	1.3 (1.6)	1.7 (2.3)	1.4 (1.8)
Antibiotics courses	1.2 (1.6)	1.3 (1.6)	1.7 (2.3)	1.3 (1.6)
Steroid courses	0.7 (1.3)	0.7 (1.4)	1.4 (2.2)	0.8 (1.4)
Out of hours visits	0.1 (0.4)	0.2 (0.5)	0.2 (0.8)	0.1 (0.5)
Attended A&E	0.1 (1.1)	0.1 (0.5)	0.2 (0.5)	0.1 (0.9)
Bed days	0.5 (3.0)	0.8 (4.0)	0.7 (2.4)	0.6 (3.3)

**Figure 2:**
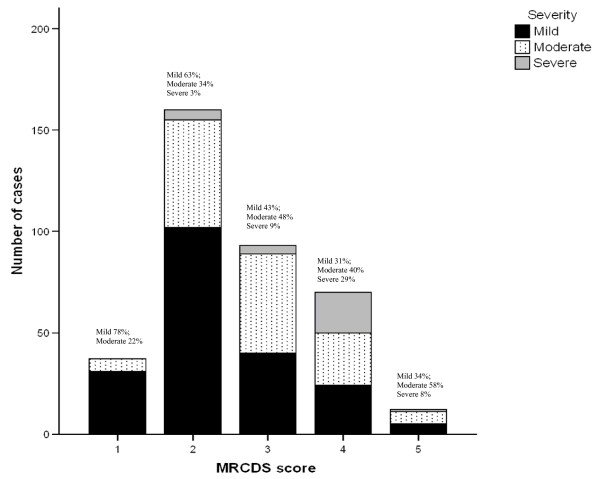
Distribution of MRC dyspnoea score for different degrees of severity of airflow obstruction.

Figure 2 shows counts of MRC dyspnoea scale scores in the COPD sample, for different degrees of severity. Results for the LINQ and the CCQ will be reported elsewhere.

In the previous two years, 93/422 (22%) patients had attended a consultant chest physician; 36/422 (9%) patients had seen a COPD specialist nurse and 3/422 (0.7%) had seen a respiratory specialist physiotherapist. In the previous five years, 187/384 (49%) of patients had had a chest x-ray, at time of diagnosis. Patients who had attended a consultant chest physician were similar to those who did not attend in respect of diagnosis and spirometry: mean FEV1% of predicted was 45.7% (SD 14.8) and 48.5% (SD 13.8) respectively (*t *= 1.68, *p *= 0.09), but had higher MRC dyspnoea scores: median 3.1 (IQR 1.3) and 2.7 (IQR 1.50), *p *= 0.002. No differences were noted between those attending and those not attending secondary care were noted with respect of age, smoking status, pack years or in the proportions that were on the recommended treatment with short and long acting anti-cholinergic, long acting beta-2-agonists inhaled steroids or mucolytics therapies.

### Management of COPD and compliance with guidelines

Data on current or recommended treatment were available for 278 of the 422 patients diagnosed with COPD (Table 4).

**Table 4:** Current and recommended treatment in patients with confirmed COPD and proportion of patients in whom a treatment was recommended who were receiving that treatment

	No. currently receiving the treatment	No. for whom the treatment was recommended	No. receiving the treatment recommended for them
	(N = 278)	(N = 278)	(N = 278)
SAAC	124 (44.6%)	109 (39.2%)	59/109 (54%)
LAB2 agonists	124 (44.6%)	80 (28.8%)	37/80 (46%)
LAAC	50 (18.0%)	53 (19.1%)	12/53 (23%)
Inhaled steroids	167 (60.0%)	48 (17.0%)	39/48 (81%)
Mucolytics	9 (3%)	144 (53.0%)	6/144 (4%)

Long acting bronchodilators were recommended for more patients than had received them and were therefore under prescribed. The NICE guidelines recommend that mucolytic therapy should be considered in patients with a chronic cough productive of sputum. Although 53% had a chronic productive cough, only 4% of these were receiving mucolytics. By contrast, 60% of patients were receiving inhaled steroids, of which only 23% met the indication of FEV1, less than 50% of predicted and two or more exacerbations per year [5]. Nine patients for whom inhaled steroids would be recommended had not received them, again highlighting the problem of inappropriate prescribing.

Practices varied substantially in prescribing long acting bronchodilators. The proportion of patients prescribed long acting beta-2-agonists ranged between 23–56% in different practices, and between 9–25% for long acting anticholinergics.

Prescribing outside of licensed indications was noted in six patients who were taking short acting anticholinergics and long acting anticholinergics therapy at the same time. Although long acting anticholinergics are only licensed for use in COPD, six patients who were found not to have COPD were on long acting anticholinergics.

Vaccination status was recorded for immunisation against pneumococcus and influenza, both of which are recommended in COPD. Influenza vaccine was up to date in 346/398 (87%) and pneumococcal vaccination was up to date in 227/380 (60%).

Data on attendance or recommendation to attend pulmonary rehabilitation were available for 372 COPD patients. Recommendations were based on the NICE guidelines which state that "Pulmonary rehabilitation should be offered to all those who consider themselves functionally disabled by COPD (usually MRC dyspnoea scale score of three and above)" [5].

Pulmonary rehabilitation was not available in North Devon, but 54/134 (40%) North Devon patients had an MRC dyspnoea scale score of three or more. None of these patients had attended pulmonary rehabilitation. In Plymouth, 121/238 (51%) had MRC dyspnoea scale score of three or more and 80/238 patients (34%) were willing, suitable and able to take part. Of these, five had an MRCDS score of less than three and 7/80 (9%) had attended rehabilitation.

## Discussion

COPD is an important disease that compromises patients' quality of life and creates a huge financial burden to the National Health Service. Our study confirms that disease management takes place largely in primary care. Guidelines suggest that an accurate diagnosis should be followed by effective treatments of stable disease and exacerbations of COPD.

Diagnostic registers have inherent problems. According to the present findings, registers include large numbers without COPD. In this study, 27% of individuals listed as having COPD were eligible for reclassification of their disease following structured clinical assessment by a trained nurse. These findings confirm the problems previously observed with application and interpretation of spirometry in primary care.

The application of reversibility testing is a controversial issue in the diagnosis of COPD in primary care [26]. Recent guidelines have suggested that reversibility testing should be used where clinically indicated to separate asthma from COPD [5,6], but there is a lack of good evidence to underpin these recommendations [26]. Reversibility testing records the change in FEV1 before and after bronchodilators and the magnitude of this change has been used to differentiate asthma from COPD [5,27].

Reversibility testing was found to change the diagnosis from that made on pre-bronchodilator spirometry in 11% of cases. This finding demonstrates the benefits of judicious use of reversibility testing based on clinical need to exclude asthma as opposed to performing reversibility testing in all cases. This study confirms that performing pre-bronchodilator spirometry alone leads to overestimation of the prevalence and severity of COPD, with the potential to cause errors in treatment. The use of pre-bronchodilator spirometry alone cannot be recommended for diagnosis and severity assessment-spirometry in primary care should be done only after administration of bronchodilators. This approach is in keeping with the current GOLD guidelines, whereas the NICE guidelines do not specify the importance of post-bronchodilator readings.

This study examined current treatment against treatment as recommended by the NICE guidelines. Under-prescribing with bronchodilators, particularly long acting agents was apparent. Long acting bronchodilators are known to improve lung function, exercise tolerance, symptoms, and quality of life, and to reduce exacerbations [6]. Furthermore, it is known that bronchodilators interact with other treatments to improve outcomes. The combination of fluticasone and salmeterol reduced decline in lung function over 3 years, [28] and outcomes in pulmonary rehabilitation were improved by tiotropium [29]. The reason why these drugs are not prescribed may be due to concerns over price, a lack of knowledge about the benefits of these medicines [19] or from an unjustified nihilistic approach to COPD management [30]. There was no evidence that this phenomenon was limited to primary care, those attending a consultant chest physician in the previous five years were no more likely to be treated according to guidelines.

This study highlights an apparent over-treatment with inhaled steroids. Only a minority of those who received inhaled steroids met the NICE criteria. In some cases, treatment with steroids may once have been not only appropriate, but also effective in reducing exacerbations until they were no longer appeared necessary. Over-treatment suggests a waste of resources and puts patients at risk of adverse effects. These findings concur with Decramer et al. [15], who revealed that 49% of GPs prescribed inhaled steroids to all of their COPD patients. The role of mucolytics is debated [31], but despite the evidence-based recommendations in NICE that mucolytics should be considered in patients with a chronic productive cough, mucolytics are seldom prescribed to such patients.

Quitting smoking improves patients' prognoses in COPD, and offering support to stop smoking reduces mortality and morbidity from COPD [1]. It is disappointing that a quarter of current smokers with COPD had not been offered practical help in terms of referral to smoking cessation service or drug therapy. While patients may not accurately report the help they have been offered, Rutschmann et al. [19] found that many physicians reported feeling uncomfortable giving smoking cessation advice, and a fifth of physicians were unaware that smoking cessation had a positive impact on life expectancy and disease progression.

Pulmonary rehabilitation is highly effective and is a cost effective intervention recommended by national and international COPD guidelines. The finding in this study was striking-in certain geographic regions pulmonary rehabilitation was not available at all and where it was available, only 9% of eligible patients had attended. Identification of patients suitable for rehabilitation is an important component of COPD assessment, as currently the proven benefits of pulmonary rehabilitation are being denied to many patients.

This study used a novel information technology system that provides a systematic and comprehensive assessment package for COPD, and facilitates the delivery of high-quality care according to guidelines. The system assesses clinical data and examines patient-centred outcomes such as quality of life and patients' perceived information needs. By providing reports to both GPs and patients it offers a new approach to COPD management in primary care. Management according to the guidelines is promoted with the potential to improve care. The system also has an educational function as GPs and nurses are given the opportunity to learn about COPD management. Given these advantages, use of the system has the potential to enable long-term improvement of COPD management. However, using a different IT-system for guideline-based management of COPD and asthma, Tierney et al. [32] reported no enhanced adherence to guidelines and no beneficial effects on patient-centred and clinical outcomes over a year. Research into the benefits of our system in terms of long-term improvement of clinical care and patient-centred outcomes is ongoing.

This study has some limitations. Although the participating practices provided a spectrum of COPD services and varied widely in their size and socio-demographic features, they may not representative as they were all in Devon and were included on the basis of their interest in the project. Furthermore, as only patients who were able to attend primary care clinics were included, those most severely affected by their COPD and those with important co-morbidities may have been under-represented.

The software system made recommendations for specific treatments based on guidelines and some recommendations were not definite statements that the treatment was required, but that the treatment should be considered, (for example mucolytics therapy). Thus the apparent under-prescribing in these situations is more difficult to interpret and may be appropriate use of therapy. A further limitation was that this was a cross-sectional study and did not assess the impact of recommendations on changing treatment or other outcomes. The actual decision by the GP to prescribe, the patients' compliance, response to treatment and duration were outside the scope of the study. The software system had not been formally validated in other studies.

## Conclusion

The present study represents one of the first studies to report the true levels of severity of COPD and the provision of appropriate treatments in primary care. The study demonstrates that compared to diagnoses made by expert nurses with the help of a standardised, guideline-based computer program, the diagnostic registers in primary care are inaccurate in 27% of cases. The role of reversibility testing was examined and it was found that pre-bronchodilator readings alone overestimated both prevalence and the severity of COPD. Reversibility testing was useful in detecting some cases where a diagnosis of asthma was considered. On this basis it is suggested that only post bronchodilator readings should be used routinely in primary care, but reversibility testing is appropriate in specific cases where asthma is suspected. The management of patients with COPD seldom followed guideline based recommendations in terms of drug treatment and pulmonary rehabilitation.

In the future, computer-based assessment systems which provide management recommendations may facilitate optimal diagnosis and treatment for patients with COPD in primary care.

## Competing interests

Dr Jones has received educational, research or travel grants from Glaxo Smith Kline; Astra Zeneca; Boehringer-Ingelheim; Pfizer; Ivax; Novartis and Altana. Dr Jones was a director of Patient Centred Software Ltd., the provider of the software used in this project.

## Authors' contributions

RJ and BS designed and supervised the study and contributed to the manuscript. MS analysed data and drafted the manuscript. MM recruited North Devon practices and carried out assessments in North Devon. DM acted as the project nurse and contributed to data preparation. All authors have read and approved the final manuscript.
